# Use of multiple laboratory tests including anti-factor Xa to optimally manage anticoagulation during ECMO

**DOI:** 10.1186/s13054-020-03145-5

**Published:** 2020-07-13

**Authors:** Patrick M. Honore, Leonel Barreto Gutierrez, Luc Kugener, Sebastien Redant, Rachid Attou, Andrea Gallerani, David De Bels

**Affiliations:** ICU Department, Centre Hospitalier Universitaire Brugmann-Brugmann University Hospital, Place Van Gehuchtenplein, 4, 1020 Brussels, Belgium

We read with great interest the recent article by Chlebowski et al., who recommend the use of multiple laboratory tests including anti-factor Xa (anti-Xa) to optimally manage anticoagulation during ECMO [1]. Anti-Xa directly measures heparin inhibition of factor Xa and is increasingly used to measure heparin effect, especially in pediatric patients [1]. Anti-Xa assay correlates better with unfractionated heparin (UFH) concentration than with activated clotting time (ACT) or activated partial thromboplastin time (aPTT) [2, 3]. The major criticism made of using anti-Xa in isolation to titrate heparin for anticoagulation is that while it is a direct measure of heparin effect, it does not represent the overall hemostatic state of the patient [1]. For example, a patient who before heparin therapy is highly prothrombotic may still be prothrombotic with what is considered to be a therapeutic effect of heparin based on anti-Xa levels [1]. We would like to make some comments. Prior to the COVID-19 pandemic, we routinely used anticoagulation for veno-arterial ECMO (VA-ECMO), but not for veno-venous ECMO (VV-ECMO) [4]. We have found the situation to be totally different in COVID-19 patients treated with VV-ECMO. We have needed to use very high doses of heparin, from 20,000 IU up to 50,000 IU, and despite those high doses, we have had several cases of thrombosis. In an attempt to avoid both bleeding and thrombosis, we have started monitoring anticoagulation in these patients with both anti-Xa and aPTT. If the anti-Xa level is within the reference range (0.3–0.7 IU/ml) but the aPTT is low, we increase the UFH, aiming for an aPTT between 50 and 70 s (according to a sliding scale). On the other hand, if the aPTT is between 50 and 70 s, but the anti-Xa is greater than 1 IU/ml, we reduce the UFH (again according to a sliding scale). By adjusting the UFH dose on the basis of a combination of two different measurements, we have aimed to improve our anticoagulation strategy and potentially reduce bleeding and thrombosis. Obviously, to confirm this, we would need to perform a randomized controlled trial. A sliding scale for UFH is easy to obtain whereas it is somewhat more difficult for anti-Xa. With this in mind, we have included our sliding scale for anti-Xa at the end of this letter (adapted from a sliding scale from the literature (Table 1) [5]).

**Table 1:** Adjustment of unfractionated heparin dose during ECMO according to anti-Xa activity and body weight. 20,000 IU UFH + 44 cc NaCl 0.9%

Anti-Xa activity	Bolus	Variation	55–64 kg	65–74 kg	75–84 kg	85–94 kg	95–104 kg	105–114 kg	115–124 kg
< 0.2	26 U/kg	↑96 U/kg/h	↑0.6 cc/h	↑0.7 cc/h	↑0.8 cc/h	↑0.9 cc/h	↑1 cc/h	↑1.1 cc/h	↑1.2 cc/h
0.20–0.29	No	↑48 U/kg/h	↑0.3 cc/h	↑0.3 cc/h	↑0.4 cc/h	↑0.4 cc/h	↑0.5 cc/h	↑0.6 cc/h	↑0.7 cc/h
0.30–0.70	No								
0.71–0.80	No	↓24 U/kg/h	↓0.1 cc/h	↓0.1 cc/h	↓0.2 cc/h	↓0.2 cc/h	↓0.2 cc/h	↓0.3 cc/h	↓0.3 cc/h
0.81–0.99	No	↓48 U/kg/h	↓0.3 cc/h	↓0.3 cc/h	↓0.4 cc/h	↓0.4 cc/h	↓0.5 cc/h	↓0.6 cc/h	↓0.7 cc/h
> 1	No	↓96 U/kg/h	↓0.6 cc/h	↓0.7 cc/h	↓0.8 cc/h	↓0.9 cc/h	↓1.0 cc/h	↓1.1 cc/h	↓1.2 cc/h

The table can be adapted: For 30,000 IU of UFH, use a correction factor of 1.5. For 40,000 IU of UFH, use a correction factor of 2. For 50,000 IU of UFH, use a correction factor of 2.5

## Authors’ response

Chlebowski MM, Baltagi S, Carlson M, Levy JH, Spinella PC

The authors agree that COVID-19 patients have increased anticoagulation needs and, as a result, multifactorial analysis of hemostasis utilizing thromboelastography (TEG) in these patients may be even more important due to the multidimensional hemostatic abnormalities that occur with COVID-19 infection [6, 7]. The current literature indicates that diffuse endothelial injury with significant inflammatory activation of platelets and reduced fibrinolysis are major contributors to the hypercoagulable state [6–8]. The use of viscoelastic assays can provide critical information regarding thrombosis risk that traditional coagulation parameters including anti-Xa, INR, PTT, and platelet levels may not provide. Recent studies show that results for these tests may be within or close to reference ranges while TEG results are consistent with profound derangements in hemostasis [6–8]. Specifically, the use of TEG strengthens the assessment of a patient’s hemostatic profile by adding information regarding clot strength, a measure of platelet function, and fibrinolysis in a whole blood sample versus other parameters, such as PTT, that are plasma-based. As a result of this data, it is important to also consider that the isolated use of anti-Xa inhibitors to anticoagulate patients with COVID-19 may be inadequate and the addition of viscoelastic assays could indicate the need for antiplatelet agents or antifibrinolytics that could be titrated carefully with serial viscoelastic testing to allow for targeted reduction in the hypercoagulable state while simultaneously reducing the risk of bleeding.

## Data Availability

Not applicable.

## Abbreviations

Anti-Xa: Anti-factor Xa
ECMO: Extracorporeal membrane oxygenation
VV-ECMO: Veno-venous extracorporeal membrane oxygenation
VA-ECMO: Veno-arterial extracorporeal membrane oxygenation
UFH: Unfractionated heparin
ACT: Activated clotting time
aPTT: Activated partial thromboplastin time
